# The relationship between HLA-B*51 subtypes, clinical manifestations and severity of Behçet’s syndrome: a large Italian cohort study

**DOI:** 10.1093/rap/rkad087

**Published:** 2023-10-17

**Authors:** Pietro Leccese, Maria Carmela Padula, Eustachio Vincenzo Santospirito, Rosa Colucci, Nancy Lascaro, Angela Anna Padula, Salvatore D’Angelo

**Affiliations:** Rheumatology Institute of Lucania (IReL) and Rheumatology Department of Lucania, San Carlo Hospital of Potenza, Potenza, Italy; Rheumatology Institute of Lucania (IReL) and Rheumatology Department of Lucania, San Carlo Hospital of Potenza, Potenza, Italy; Tissue Typing Laboratory C.R.T. Basilicata, Madonna delle Grazie Hospital, Matera, Italy; Tissue Typing Laboratory C.R.T. Basilicata, Madonna delle Grazie Hospital, Matera, Italy; Rheumatology Institute of Lucania (IReL) and Rheumatology Department of Lucania, San Carlo Hospital of Potenza, Potenza, Italy; Rheumatology Institute of Lucania (IReL) and Rheumatology Department of Lucania, San Carlo Hospital of Potenza, Potenza, Italy; Rheumatology Institute of Lucania (IReL) and Rheumatology Department of Lucania, San Carlo Hospital of Potenza, Potenza, Italy

**Keywords:** Behçet’s syndrome, HLA-B*51, genotyping, subtypes

## Abstract

**Objectives:**

Behçet’s syndrome (BS) is a chronic multisystemic inflammatory disorder of unclear aetiology. The predominant BS susceptibility locus was identified within HLA-B*51. HLA-B*51 subtypes were previously studied as disease susceptibility markers. Few data are now available about the relationship between B*51 subtypes and clinical phenotype. The aim of this study was to genotype HLA-B*51 subtypes in a series of Italian BS patients and to test the association with clinical manifestations and disease severity (Krause’s index).

**Methods:**

HLA-B*51 subtype genotyping for 63 alleles (B*51:01–B*51:63) was performed by PCR after DNA extraction from whole blood of BS patients. The correlation of disease clinical manifestations and severity (Krause’s index) with the HLA-B*51 allele and its subtypes was analysed.

**Results:**

We enrolled 241 (140 male and 101 female) BS patients, and HLA-B*51 frequency was 62.7% (151 of 241). One hundred and eight of the HLA-B*51-positive patients carried the B*51:01 subtype (108 of 151, 71.5%), 39 of 151 (25.8%) the B*51:08 subtype, 2 of 151 (1.3%) the B*51:02 subtype, 1 of 151 (0.7%) the B*51:05 subtype, and 1 of 151 (0.7%) the B*51:07 subtype. We found that ocular involvement was statistically associated with HLA-B*51 positivity and with B*51:01 and B*51:08 subtypes (*P* < 0.05). We also found that disease severity was higher in HLA-B*51-positive patients than in negative patients, but without statistical significance (median Krause’s index 5.1 *vs* 4.1, *P* > 0.05).

**Conclusion:**

Here, we confirm a high frequency of the HLA-B*51 allele in our group of BS patients. B*51:01 and B*51:08 were found to be the most common subtypes, and an association of both subtypes with ocular involvement was also underlined.

Key messages

- Genotyping of a large series of Italian Behçet’s syndrome patients showed high frequency of HLA-B*51.
- B*51:01 and B*51:08 were the most common subtypes in our group of patients.
- B*51:01 and B*51:08 were associated with ocular involvement.

## Introduction

Behçet’s syndrome (BS) is a systemic vasculitis of unknown origin characterized by typical ocular and mucocutaneous manifestations and by a chronic-relapsing course. The disorder shows a particular geographical distribution corresponding to the historical Silk Road, probably attributable to the association of environmental factors and genetic markers of susceptibility. Among the different aetiological factors studied so far, the HLA-B*51 antigen seems to be more closely linked to the disease susceptibility [1, 2]. HLA molecules are controlled by genes located at the terminal region at band p21.3 on chromosome 6, whose products control many functions, including the regulation of immune responses and inflammation [3]. The role of HLA-B*51 in the pathogenesis of BS is still somewhat unclear. It is known that the HLA-B*51 is implicated in the presentation of endogenous antigens and involved in the abnormal T-lymphocyte activity and neutrophil function observed in BS patients [4, 5]. HLA-B*51-transgenic mice showed enhanced neutrophil function, although they did not develop a BS phenotype [6]. Given that the role of HLA-B*51 is to present endogenous peptides to CD8^+^ T cells, the lack of the disease symptoms in the mouse model can be related to the absence of a triggering microbial or injury-related peptide that would activate the CD8^+^ T cells [7]. Ohno *et al.* [8] have suggested that BS became prevalent along the Silk Route owing to the distribution of HLA-B*51 through interactions between the autochthonous population with the old nomadic tribes or the Turks. The association of HLA-B*51 with BS partly explains the geographical distribution of the disease. In this context, the Sardinian population is a genetic isolate where the gene allele and haplotype distribution are highly conserved owing to geographical isolation and endogamy [6]. In the past few years, the distribution of different HLA-B*51 subtypes has been analysed in several studies and summarized in two meta-analyses [9, 10].

So far, HLA-B*51:01 has been found to be the most frequent allele associated with BS worldwide, in particular in different ethnic groups, such as Greek [11, 12], Spanish [13], Italian [6, 14, 15], Saudi Arabian [16], Iranian [17], German [18], Turkish [18, 19] and Japanese [20] patients, but not in the Israeli [21] population. HLA-B*51:08 was another frequent subtype, with a lower percentage compared with HLA-B*51:01, ranging from 2.2% (Italian BS population) [6] to 22.4% (Greek BS population) [12]. In a previous study by our group, 50% of BS patients showed the B*51:01 subtype, while the frequency of B*51:08 was 11.8% [15].

The number of identified B*51 subtypes has been increasing continuously. A novel HLA-B*51 allele (HLA-B*51:94), differing from HLA-51:01 by a nucleotide exchange (C>T at 403 position) in exon 3, has been described recently [22]. In the Brazilian population, another *de novo* variation, HLA-B*51:151, was reported [23], and the novel allele HLA-B*51:220, generated by a gene-conversion event, was also identified recently [24].

So far, only a few small studies have evaluated the relationship between HLA-B*51 subtypes and clinical findings in BS. The objective of this study was to investigate the association between clinical features and severity if BS with HLA-B51 status and its subtypes by genotyping 63 different subtypes (B*51:01–B*5163) in a large series of BS patients.

## Methods

We enrolled consecutive patients with BS originating and living in Italy, seen at the outpatient clinic of the Rheumatology Department of Lucania (Basilicata, Italy). All BS patients met the International Study Group (ISG) classification criteria [25]. Patients’ demographic and clinical data were collected from their medical records. Disease severity was assessed according Krause’s index [26]. Blood samples were collected in our laboratory. All subjects gave their informed consent to this study, which was conducted according to the Good Clinical Practices (GCP) and the Declaration of Helsinki. The Regional Ethics Committee [Comitato Etico Unico Regionale (CEUR)] approved the study (permit number: 705/2017).

DNA was isolated from blood leucocytes by standard methods. Genotyping of HLA-B*51 subtypes for 63 alleles (B*51:01–B*5163) was performed by the PCR method with a GeneAmp PCR System 9700 (Applied Biosystems, Foster City, CA, USA) using the primer mixes included in the kit for SSP subtyping.

We used χ^2^ or Fisher’s exact tests for comparing HLA-B*51 subtypes with both BS clinical manifestations and disease severity score (Krause’s score). The odds ratio (OR) with 95% confidence interval (CI) was calculated to assess the strength of associations. A difference was considered significant if the *P*-value was < 0.05. SPSS v.13 software (SPSS, Chicago, IL, USA) for Windows was used for statistical analyses.

## Results

This study was conducted on 241 (140 male and 101 female) patients with BS having a mean age of 45.2 ± 13.4 years. The frequency of HLA-B*51 was 62.7% (151 of 241). By considering only the allele frequencies among HLA-B*51-positive subjects, 108 of 151 (71.5%) patients carried the B*51:01 subtype, 39 of 151 (25.8%) the B*51:08 subtype, 2 of 151 the B*51:02 subtype (1.3%), 1 of 151 the B*51:05 subtype (0.7%), and 1 of 151 the B*51:07 subtype (0.7%) (Table 1).

**Table 1.** HLA-B*51 subtype frequencies in Italian Behçet’s syndrome patients

Subtype	BS patients [*n* (%)]
	*n* = 241
B*51	151 (62.7)
B*51:01	108 (44.8)
B*51:02	2 (0.8)
B*51:03	0 (0.0)
B*51:04	0 (0.0)
B*51:05	1 (0.4)
B*51:06	0 (0.0)
B*51:07	1 (0.4)
B*51:08	39 (16.2)
B*51:09-63	0 (0.0)

BS: Behçet’s syndrome.

No significant difference was found for sex or for disease clinical manifestations between B*51-positive and negative groups, except for posterior uveitis. This condition was found in 46% of HLA-B*51-positive patients (70 of 151) and in 28% of HLA-B*51-negative patients (25 of 90), (*P* < 0.05). HLA-B*51 positivity conferred a risk of posterior uveitis about two times higher than in the case of HLA-B*51 negativity (OR 2.25; CI: 1.28–3.94; *P* < 0.05; Table 2).

**Table 2.** Frequency of clinical manifestations of Behçet’s syndrome according to the HLA-B*51 positivity

Manifestation	B*51-positive	B*51-negative	*P*-value	Odds ratio	95% CI
	(*n* = 151)	(*n* = 90)			
Oral ulcers	151 (100)	90 (100)	0.90	1.68	0.00–NA
Genital ulcers	84 (55.6)	59 (65.6)	0.1291	0.66	0.28–1.13
Papulopustular lesions	114 (75.5)	60 (66.7)	0.1389	1.54	0.87–2.74
Erythema nodosum	78 (51.7)	42 (46.7)	0.4537	1.22	0.72–2.06
Folliculitis	27 (17.9)	14 (15.6)	724.00	1.18	0.58–2.39
Pathergy test positivity	12 (7.9)	9 (10)	0.5846	0.78	0.31–1.92
Anterior uveitis	45 (29.8)	25 (27.8)	0.7378	1.10	0.62–1.97
Posterior uveitis	70 (46.4)	25 (27.8)	**0.0043**	2.25	1.28–3.94
Arthritis	44 (29.1)	23 (25.6)	0.5481	1.20	0.66–2.16
CNS involvement	20 (13.2)	11 (12.2)	0.8185	1.100	0.50–2.41
Superficial venous thrombosis	17 (11.3)	12 (13.3)	0.6320	0.82	0.37–1.82
Deep venous thrombosis	7 (4.6)	7 (7.8)	0.3131	0.58	0.20–1.70
Arterial involvement	4 (2.6)	1 (1.1)	0.4178	2.42	0.27–22.01
Gastrointestinal involvement	17 (11.3)	9 (10)	0.7607	1.14	0.49–2.68
Fever	54 (35.8)	36 (40)	0.5106	0.84	0.49–1.43
Fatigue	37 (24.5)	27 (30)	0.3500	0.76	0.42–1.36

Bold text indicates statistical significance.

**Figure 1.**
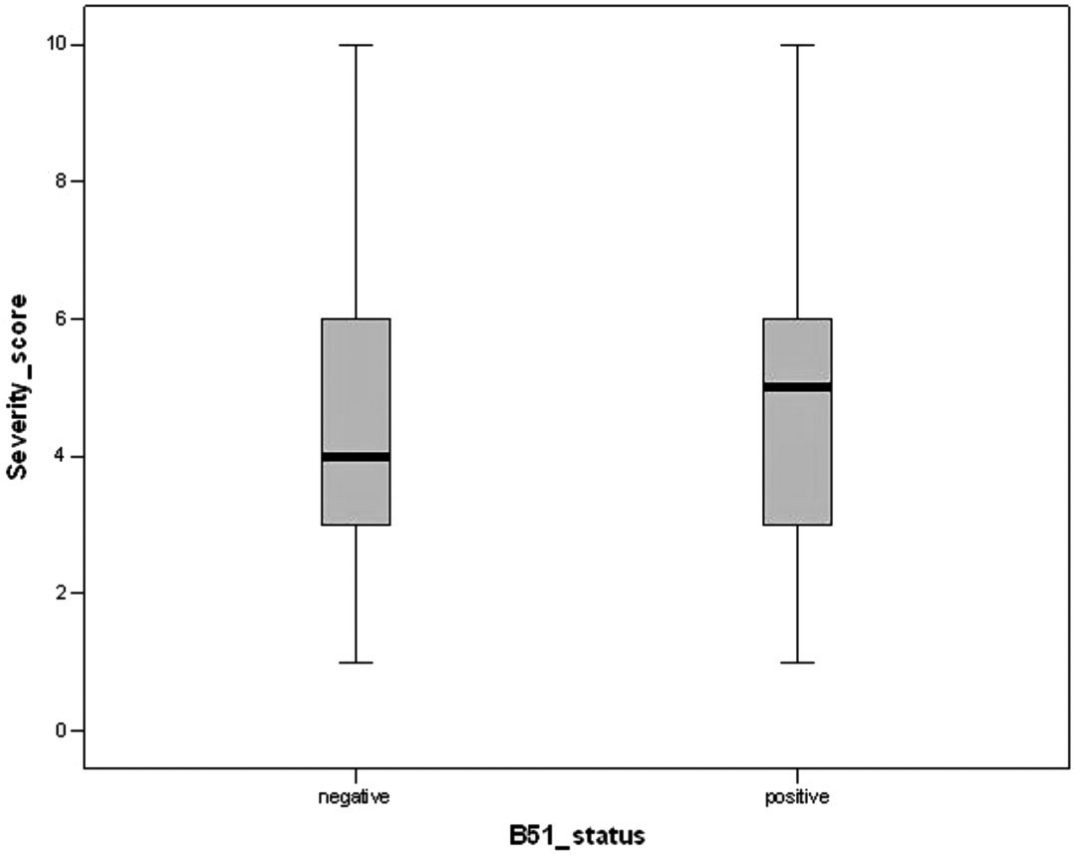
Relationship between Krause’s severity index and B*51 status

We studied the disease severity according to the HLA-B*51 status (Fig. 1). We observed a higher median value for patients with the presence of the allele compared with patients without the allele (median Krause’s index 5.1 for positive patients *vs* 4.1 for negative patients without statistical significance; *P* > 0.05).

We also stratified the clinical manifestations for the most common subtypes (HLA-B*51:01 and HLA-B*51:08) and found that anterior uveitis was less common in the case of B*51:01 positivity than in the case of B*51:08 positivity (24.1 and 46.1%, respectively; *P* < 0.05; OR 0.37; CI 0.17–0.80; Table 3). A statistically significant higher frequency of anterior uveitis was also found in the group with B*51:08 compared with the B*51-negative patients (46.2 *vs* 27.8%, respectively; *P* < 0.05). B*51:01 positivity was identified as a risk marker for posterior uveitis, conferring a risk of posterior uveitis about two times higher than HLA-B*51 negativity (*P* < 0.05; OR 1.93; CI: 1.06–3.51).

**Table 3.** Clinical manifestations of Behçet’s syndrome in HLA-B*51:01- and HLA-B*51:08-positive patients

Manifestation	B*51:01-positive	B*51:08-positive	B*51-negative	***P*-value** ^a^	***P*-value** ^b^	***P*-value** ^c^
	(*n* = 108)	(*n* = 39)	(*n* = 90)	OR (95% CI)	OR (95% CI)	OR (95% CI)
Oral ulcers	108 (100)	39 (100)	90 (100)	0.8123	0.9675	0.8475
				2.77 (0.00–NA)	1.20 (0.00–NA)	0.43 (0.00–NA)
Genital ulcers	65 (60.2)	19 (48.7)	59 (65.6)	0.2148	0.4367	0.7753
				1.59 (0.76–3.32)	0.79 (0.44–1.42)	1.13 (0.50–2.53)
Papulopustular lesions	84 (77.8)	27 (69.2)	60 (66.7)	0.2874	0.0805	0.7753
				1.56 (0.69–3.52)	1.75 (0.93–3.29)	1.13 (0.50–2.53)
Erythema nodosum	58 (53.7)	19 (31.1)	42 (46.7)	0.5931	0.2535	0.8303
				1.22 (0.59–2.54)	1.39 (0.79–2.43)	1.09 (0.51–2.30)
Pathergy test positivity	10 (9.3)	2 (5.1)	9 (10)	0.4193	0.92	0.3629
				1.89 (0.39–9.02)	0.92 (0.36–2.37)	0.49 (0.10–2.36)
Anterior uveitis	26 (24.1)	18 (46.2)	25 (27.8)	**0.0099**	0.5529	**0.0420**
				0.37 (0.17–0.80)	0.82 (0.44–1.56)	2.23 (1.02–4.86)
Posterior uveitis	46 (42.6)	22 (56.4)	25 (27.8)	0.1380	**0.0304**	**0.0019**
				0.57 (0.27–1.20)	1.93 (1.06–3.51)	3.36 (1.54–7.36)
Arthritis	32 (29.6)	11 (28.2)	23 (25.6)	0.8669	0.5239	0.7537
				1.07 (0.48–2.41)	1.23 (0.65–2.30)	1.14 (0.49–2.66)
CNS involvement	22 (20.4)	6 (15.4)	11 (12.2)	0.4967	0.1256	0.6258
				1.41 (0.52–3.78)	1.84 (0.84–4.03)	1.31 (0.45–3.82)
Superficial venous thrombosis	12 (11.1)	5 (12.8)	12 (13.3)	0.7748	0.6333	0.9370
				0.85 (0.28–2.59)	0.81 (0.35–1.91)	0.96 (0.31–2.92)
Deep venous thrombosis	6 (5.6)	0 0	7 (7.8)	0.1549	0.5296	0.0850
				22.88 (0.04–NA)	0.70 (0.23–2.16)	0.03 (0.00–NA)
Arterial involvement	4 (3.7)	0 0	1 (1.1)	0.2624	0.2469	0.6271
				14.96 (0.03–NA)	3.42 (0.38–31.19)	0.23 (0.00–NA)
Gastrointestinal involvement	13 (12.0)	4 (10.3)	9 (10)	0.7657	0.6497	0.9646
				1.20 (0.37–3.92)	1.23 (0.50–3.03)	1.03 (0.30–3.56)
Fever	34 (31.5)	18 (26.2)	36 (40)	0.1005	0.2119	0.5153
				0.54 (0.25–1.13)	0.69 (0.38–1.24)	1.29 (0.60–2.74)
Fatigue	20 (16)	8 (44)	27 (30)	0.7857	0.0587	0.2657
				0.88 (0.35–2.20)	0.53 (0.27–1.03)	0.60 (0.25–1.48)

Bold text indicated statistical significance.

a B*51:01-positive *vs* B*51:08-positive.

b B*51:01-positive *vs* B*51-negative.

c B*51:08-positive *vs* B*51-negative.

OR: odds ratio.

## Discussion

In the present study, we evaluated the frequency of HLA-B*51 subtypes in 241 patients with BS originating and living in Italy, where the disease is rare. These subjects were genotyped for HLA-B*51 allele and for a wide number of its subtypes (63 alleles) in order to investigate the relationship between both HLA-B*51 status and HLA-B*51 subtypes with the clinical manifestations of BS and with the disease severity. No similar studies are currently available in literature.

HLA-B*51 was found in 62.7% of cases, with a higher disease severity in the presence of the allele than in its absence. In the genotyping of HLA-B*51 subtypes, HLA-B*51:01 was the most common allele, as reported in previous studies by other groups who analysed the distribution of HLA-B*51 subtypes in Italy [6, 14]. In particular, we found HLA-B*51:01 in 44.8% of cases and HLA-B*51:08 in 16.2%. B*51:02, B*51:05 and B*51:07 were the other subtypes observed, each in only one patient, respectively.

Our data confirmed the distribution of HLA-B*51 alleles that we found in our previous study investigating the role of the allele and its subtypes in disease susceptibility: HLA-B*51 frequency was 64.5% in BS patients and 16.9% in controls [15]. Half of the patients showed the B*51:01 subtypes, while 11.8% of them were found to be B*51:08-positive. Both subtypes were statistically significantly higher in BS group than in controls. Kera *et al.* [14] also performed a B*51 subtype analysis in 21 Italian patients, 15 of whom (71.4%) were B*51-positive. Of these 21 patients, 11 were B*51:01-positive (52.4%) and 4 B*51:08-positive (19.0%).

A study on the haplotypic distribution of HLA in Sardinia showed that the frequency of HLA-B*51 was equal to 42.2%, while a frequency of 14.2% was observed within the healthy control group [5]. By comparison with our data, a slightly higher percentage was found in our samples for B*51:01 and B*51:08 positivity.

The distribution of the B*51:01 subtype in our series was similar to that found in Germany [18] (44.8 *vs* 42.2%, respectively), and the percentage of B*51:08 subtype was found to be similar to that found in another Italian investigation (19.0%) [14], and in Israeli (12.5%) and Turkish (12.0%) [18] studies (Table 4).

**Table 4.** Frequency of HLA-B*51 and frequency of HLA-B*51 subtypes in several populations

Study population	BS (*n*)	HC (*n*)	Subtype analysis	B*51 frequency		B*51 subtype frequency		
				BS [*n* (%)]	HC [*n* (%)]	Subtype	BS [*n* (%)]	HC [*n* (%)]
Greek Mizuki *et al.* (1997) [11]	31	30	51:01–51:07	25/31 (80.6)	8/30 (26.7)	51:01	25/31 (80.6)	8/30 (26.7)
Spanish Gonzalez-Escribano *et al.* (1998) [13]	57	NA	51:01 51:02 51:07 51:08	21/57 (36.8)	25/NA	51:01	18/57 (31.6)	21/NA
						51:02	0/57 (0.0)	1/NA
						51:07	0/57 (0.0)	1/NA
						51:08	3/57 (5.2)	2/NA
Italian Kera *et al.* (1999) [14]	21	28	51:01–51:09	15/21 (71.4)	5/28 (17.8)	51:01	11/21 (52.4)	5/28 (17.8)
						51:08	4/21 (19.0)	0/28 (0.0)
Italian Leccese *et al.* (2009) [15]	152	320	51:01–51:63	98/152 (65.5)	54/320 (16.9)	51:01	76/152 (50.0)	49/320 (15.3)
						51:02	2 (1.3)	0/320 (0.0)
						51:05	1 (0.7)	1 (0.3)
						51:07	1 (0.7)	3 (0.9)
						51:08	18 (11.8 )	1 (0.3)
Saudi Arabian Yabuki *et al.* (1999) [16]	13	18	51:01–51:09	10/13 (76.9)	4/18 (22.2)	51:01	9/13 (69.2)	4/18 (22.2)
						51:08	1/13 (10.0)	0/18 (0.0)
Iranian Mizuki *et al.* (2001) [17]	58	44	51:01–51:10	36/58 (62.1)	14/44 (31.8)	51:01	33/58 (56.9)	13/44 (29.5)
						51:08	3/58 (5.2)	1/44 (2.3)
Israeli Paul *et al.* (2001) [21]	24	18	51:01 51:02 51:04 51:08	13/24 (54.2)	11/18 (61.1)	51:01	8/24 (33.3)	10/18 (55.5)
						51:02	0/24 (0.0)	1/18 (0.6)
						51:04	2/24 (8.3)	0/18 (0.0)
						51:08	3/24 (12.5)	0/18 (0.0)
German Kotter *et al.* (2001) [18]	33	325	51:01 51:05 51:07 51:08	16/33 (48.5)	61/325 (18.8)	51:01	14/33 (42.4)	57/325 (17.5)
						51:05	0/33 (0.0)	1/325 (0.3)
						51:07	0/33 (0.0)	1/325 (0.3)
						51:08	2/33 (6.1)	2/325 (0.6)
Turkish Kotter *et al.* (2001) [18]	92	93	51:01 51:05 51:07 51:08	64/92 (69.6)	23/93 (24.7)	51:01	52/92 (56.5)	20/93 (21.5)
						51:05	1/92 (1.1)	0/93 (0.0)
						51:07	0/92 (0.0)	1/93 (1.1)
						51:08	11/92 (12.0)	2/93 (2.2)
Greek Mizuki *et al.* (2002) [12]	58	41	51:01–51:10	44/58 (75.9)	9/41 (22.0)	51:01	34/58 (58.6)	9/41 (22.0)
						51:08	13/58 (22.4)	0/41 (0.0)
Japanese Mizuki *et al.* (2001) [20]	96	132	51:01–51:04	57/96 (59.4)	18/132 (13.6)	51:01	56/96 (58.3)	18/132 (13.6)
						51:02	1/96 (1.0)	0/132 (0.0)
Italian (Sardinian) Piga *et al.* (2012) [6]	45	120	51:01 51:08	19/45 (42.2)	17/120 (14.2)	51:01	18/45 (40.0)	17/120 (14.2)
						51:08	1/45 (2.2)	0/120 (0.0)
Turkish Demirseren *et al.* (2014) [19]	51	44	51:01 51:09 51:22	36/51 (70.6)	6/44 (13.6)	51:01	35/51 (68.6)	6/44 (13.6)
						51:09	11/51 (21.5)	3/44 (6.8)
						51:22	9/51 (17.6)	0/44 (0.0)

BS: Behçet’s syndrome; HC: healthy controls.

The distribution of HLA-B*51 subtypes was characterized by wide heterogeneity, and the phenotypic effect also depends on polymorphisms of the endoplasmic reticulum amino peptidase gene (*ERAP1*) owing to the epistatic interaction between HLA molecules and ERAP1 [27–29]. The B*51:08 subtype differs from B*51:01 by the glutamic acid to valine amino acid substitution at position 152 and by the leucine to aspartic acid amino acid substitution at codon position 156. The two amino acids are within pocket E in the HLA molecule [12]. Mizuki and collaborators also reported that asparagine at position 63 and phenylalanine at position 67 in B*51:01 determined the genetic susceptibility to BS [12]. These two amino acids are shared by B*51:01, B*51:02 and B*51:08 but not by B*51:07 [24], hence this last subtype was considered not to be associated with BS risk [12]. The B*51:02 subtype differs from B*51:01 by a single amino acid substitution of tyrosine for histidine at residue 171, which is a conserved amino acid involved in a strong hydrogen bound within pocket A of the HLA-B*51 molecule [30].

Regarding the relationship between HLA-B*51 and clinical manifestations of BS, we recognized an association between HLA-B*51 and eye involvement (posterior uveitis). The meta-analysis by Maldini *et al.* [10] also showed that HLA-B*51 was associated with a higher prevalence of eye involvement and with genital aphthosis, in addition to a decreased prevalence of gastrointestinal involvement in BS.

Horie *et al.* [31] reported a strong correlation between HLA-B*51 and ocular lesions, in particular, in East-Eurasian and Middle-Eurasian, but not in West-Eurasian areas. An association between HLA-B*51 positivity and ocular involvement in patients with BS was also described recently by Shenavandeh *et al.* [32].

We also found that ocular involvement was associated with B*51:01 and B*51:08 subtypes. A statistically significant higher frequency of anterior uveitis was found in the group with B*51:018 compared with both B*51:01-positive patients (46.2 *vs* 24.1%, respectively; *P* < 0.05) and B*51-negative patients (46.2 *vs* 27.8%, respectively; *P* < 0.05). These results strengthened the association between ocular involvement with HLA-B*51 allele and both B*51:01 and B*51:08 most common subtypes. Although both B*51:01 and B*51:08 subtypes are associated with posterior uveitis compared with B*51-negative patients, no differences were found between the frequency of B*51:01 (42.6%) and B*51:08 (52.4%) positivity in the case of posterior uveitis, while B*51:08 was associated with anterior uveitis, suggesting a milder disease. No sex differences were found for HLA-B*51 status, according to a previous study by our group [33]. We also evaluated the relationship between HLA-B*51 and its subtypes with the disease severity (Krause’s index). Interestingly, a trend of high disease severity in HLA-B*51-positive patients was found.

## Conclusions

Our data confirm the high prevalence of B*51 in Italian patients with BS, confirming its association with disease severity. This study evaluated, for the first time, the correlation between B*51 subtypes and disease clinical manifestations by recruiting a large series of BS patients and by analysing a higher number of alleles. We found an association between B*51 and ocular involvement, in particular with poster uveitis. In addition, B*51:01 and B*51:08 subtypes were associated with eye lesions.

## Data Availability

The data presented in this study are available on request from the corresponding author.
